# Reversible Control of the Mn Oxidation State in SrTiO_3_ Bulk Powders

**DOI:** 10.3389/fchem.2019.00353

**Published:** 2019-05-22

**Authors:** Haneen Mansoor, William L. Harrigan, Keith A. Lehuta, Kevin R. Kittilstved

**Affiliations:** Department of Chemistry, University of Massachusetts Amherst, Amherst, MA, United States

**Keywords:** electron paramagnetic resonance, oxidation state, manganese, strontium titanate, inorganic materials

## Abstract

We demonstrate a low-temperature reduction method for exhibiting fine control over the oxidation state of substitutional Mn ions in strontium titanate (SrTiO_3_) bulk powder. We employ NaBH_4_ as the chemical reductant that causes significant changes in the oxidation state and oxygen vacancy complexation with Mn^2+^ dopants at temperatures <350°C where lattice reduction is negligible. At higher reduction temperatures, we also observe the formation of Ti^3+^ in the lattice by diffuse-reflectance and low-temperature electron paramagnetic resonance (EPR) spectroscopy. In addition to Mn^2+^, Mn^4+^, and the Mn^2+^ complex with an oxygen vacancy, we also observe a sharp resonance in the EPR spectrum of heavily reduced Mn-doped SrTiO_3_. This sharp signal is tentatively assigned to surface superoxide ion that is formed by the surface electron transfer reaction between Ti^3+^ and O_2_. The ability to control the relative amounts of various paramagnetic defects in SrTiO_3_ provides many possibilities to study in a model system the impact of tunable dopant-defect interactions for spin-based electronic applications or visible-light photocatalysis.

## Introduction

The oxide SrTiO_3_ is a classic perovskite-type member of the valuable ABO_3_ semiconductor family. The promising properties such as a large tunable dielectric constant, structural phase transitions, superior charge storage capacity and tunable electronic structure have made SrTiO_3_ an exciting candidate for a wide range of multifunctional applications (Weaver, 1959; Faughnan, 1971; Mattheiss, 1972; Müller and Burkard, 1979; Kamalasanan et al., 1993). Although in ambient conditions it exhibits a wide band gap and low electron mobility, introducing impurity dopants and intrinsic defects radically influence the conductivity and optical properties of the host material (Wild et al., 1973; Kozuka et al., 2010). The function of a semiconductor is intimately related to the chemistry and physics of native and targeted defects. The rich defect chemistry enabled by native oxygen vacancies (*V*_O_) in semiconductors such as SrTiO_3_, PbTiO_3_, and BaTiO_3_ has been correlated with numerous functions including ferroelectricity, visible-light photocatalysis, and multiferroics. These *V*_O_ defects can donate up to two electrons to the host lattice. Transition metal dopants may also impart additional functionality that result from partially-filled d-orbitals. For example, Cr^3+^ dopants, and Rh^3+^ dopants in SrTiO_3_ can reduce protons to generate H_2_ gas using visible light that creates an oxidized dopant ion and a conduction band electron, *e*_cb_ (Ishii et al., 2004; Sasaki et al., 2009; Kato et al., 2013). However, undesirable defects such as Cr^6+^ can form to maintain charge neutrality, but limit the photochemical efficiency by serving as a trap for the *e*_cb_. These types of high-valent defects can be removed by either post-synthetic annealing under reducing atmospheres (Zuo et al., 2010; Tan et al., 2014; Lehuta and Kittilstved, 2016), co-doping the host lattice with additional *n*-type dopants (Chan et al., 1981; Kato and Kudo, 2002; Wang et al., 2014), irradiating with UV light (Wang et al., 2006), or applying a large electrical bias (La Mattina et al., 2008). Of these, the only potentially “green” reduction source could be UV irradiation from the sun. However, we note that the reported photoreduction step using a 400 W Hg-lamp in Cr:SrTiO_3_ powders was of the order of tens of hours. The realization of a fast, low-energy method to modulate the oxidation state of transition-metal dopants in SrTiO_3_ and related metal oxide semiconductors could impact various fields such as visible-light photocatalysis, sensing, and spin-based electronics. To this end, recent studies on the photodoping of colloidal Cr:SrTiO_3_ nanocrystals show promise (Harrigan and Kittilstved, 2018).

We previously studied the effect of a relatively low-temperature NaBH_4_ reduction reaction on the oxidation state of Cr dopants in SrTiO_3_ and related Sr_2_TiO_4_ bulk powders (Lehuta and Kittilstved, 2016; Lehuta et al., 2017). In those studies, we observed an order of magnitude *increase* in the Cr^3+^ concentration by EPR spectroscopy that we attributed to the reduction of EPR-silent high-valent Cr^4+^ and Cr^6+^ ions. The increase in the Cr^3+^ concentration in *n*-type SrTiO_3_ presents an interesting scenario where the Cr^3+^ ion has a dual role of being an electron donor and a paramagnetic ion (*S* = 3/2). In addition, these observed changes are quantitatively reversible upon annealing the powders in air.

An isoelectronic analog of Cr^3+^ is Mn^4+^, which is known to also occupy the Ti^4+^-site in SrTiO_3_. Although additional defects are required to maintain charge neutrality in Cr^3+^:SrTiO_3_, Mn^4+^ in the B-site of SrTiO_3_ is an isovalent dopant. Amongst the transition-metal doped oxides, Mn:SrTiO_3_ has recently received extensive attention due to its complex and unique behavior than intrinsic SrTiO_3._ The concurrent doping of Mn and oxygen vacancies in SrTi_1−x_Mn_*x*_O_3−δ_ is reported to promote ferromagnetic ordering, dielectric permittivity and possible metallic behavior (Savinov et al., 2008; Choudhury et al., 2011, 2013; Middey et al., 2012; Thanh et al., 2014). These observations make nonstoichiometric Mn:SrTiO_3_ a highly attractive candidate for spin-based electronics applications. Although not completely understood, the results are attributed to the interplay of redox-active Mn ions and the intrinsic charge compensating defects. In this regard, quantitative research is challenging due to a lack of experimental control over the interactions, and the complexity of Mn ions present in multiple oxidation states. Herein we report on the nature of the oxidation state of Mn ions and associated defect centers in bulk Mn:SrTiO_3_ powders. We utilized various dopant-specific spectroscopic probes to elucidate the Mn oxidation state including EPR and diffuse-reflectance spectroscopies. We extend the use of NaBH_4_ as a solid-state reductant to monitor changes in the three, unique Mn-related species as well as oxygen-related defects and “self-doped” Ti^3+^ ions. Comparison to other studies of reduced Mn:SrTiO_3_ and noticeable absences of certain EPR-active Mn-centers is also discussed. We also observe a new signal in reduced samples that we attribute to superoxide anions, ${\text{O}}_{2}^{-}$.

## Materials and Methods

### Chemicals

TiO_2_ (>99.5%, Aeroxide P25 powder, Acros Organics), Sr(NO_3_)_2_ (>99%, Acros Organics), Mn(NO_3_)_2_·4H_2_O (analytical grade, Acros Organics), NaBH_4_ (≥98%, white powder, MP Biomedical), MgO (Fisher Science Education), and ethanol (200 proof, ACS/USP grade, Pharmco-Aaper) were used as received.

### Synthesis of Bulk Mn-Doped SrTiO_3_

Bulk powders of SrTi_1−x_Mn_*x*_O_3−δ_ (abbreviated Mn:SrTiO_3_) were synthesized by a conventional solid-state reaction method, where *x* is the nominal concentration of Mn (*x* = 0.001) and δ is the concentration of oxygen vacancies. Briefly, Sr(NO_3_)_2_, Mn(NO_3_)_2_·4H_2_O, and TiO_2_ were mixed in the desired stoichiometry and ground with a mortar and pestle for about 10 min. The mixture was then transferred to a porcelain combustion boat and placed in the center of a tube furnace inside a quartz insert. The reaction mixture was heated in air for 6 h at 1,000°C, reground for 10 min, then heated again for an additional 16 h at 1,000°C.

### NaBH_4_ Reductions and Reoxidation

Chemical reductions of the bulk powders were carried out using a modified version of reduction previously described by our group for Cr:SrTiO_3_ (Lehuta and Kittilstved, 2016). For each reduction, an amount of powder was mixed in a 1:1 mole ratio with NaBH_4_ using a mortar and pestle for 5 min and then placed in a porcelain combustion boat in the middle of a quartz insert in a tube furnace. The atmosphere in the quartz insert was continuously purged by a controlled flow of Ar gas monitored by a rotameter (Matheson 7300). The samples were heated at temperatures ranging from 300 to 425°C in 25°C increments under Ar flow for 30 min. After reducing, samples were cooled under Ar to room temperature, washed and centrifuged alternately with deionized water and ethanol to ensure complete removal of NaBH_4_. After washing, samples were dried in an oven at 100°C for 2 h.

Reoxidation was performed by aerobically annealing the reduced samples until the physical color of the sample reversed to Mn:SrTiO_3_ as-prepared sample.

### Characterization

Powder X-ray diffraction (XRD) patterns were collected at room temperature using a Bragg-Brentano configuration with Cu K-α source (Rigaku SmartLab SE Diffraction System with cross-beam optics and D/Tex 250 Ultra 1D Si strip detector). X-band quantitative EPR spectra were collected at room temperature in 4 mm quartz EPR tubes (Wilmad-Glass) in a double rectangular resonator cavity (Bruker Elexsys E-500 with ER 4105DR cavity). Room temperature quantitative EPR spectra were collected consecutively on chemically perturbed samples (either oxidized or reduced) and an as-prepared sample using identical sample placement and instrument settings (Eaton et al., 2010). The resonance field positions in the EPR spectra for each paramagnetic Mn center were simulated using the “resfields” function in EasySpin using the reported EPR parameters from literature and referenced below (Stoll and Schweiger, 2006). Low-temperature X-band EPR spectra were measured at 77 K on powders using the perpendicular mode of a dual-mode resonator cavity with a quartz finger dewar insert (Bruker Elexsys E-500 with ER-4116 cavity) ensuring the sample height exceeded the cavity height for quantitative analysis. Diffuse-reflectance spectra were collected with an integrating sphere (Ocean Optics ISP-REF) coupled by fiber optics to a CCD-based spectrophotometer (Ocean Optics USB2000+ VIS-NIR). The optical density between the absorption minimum and the absorption at 320 nm was adjusted by diluting the powders with MgO.

## Results and Discussion

**Figure 1:**
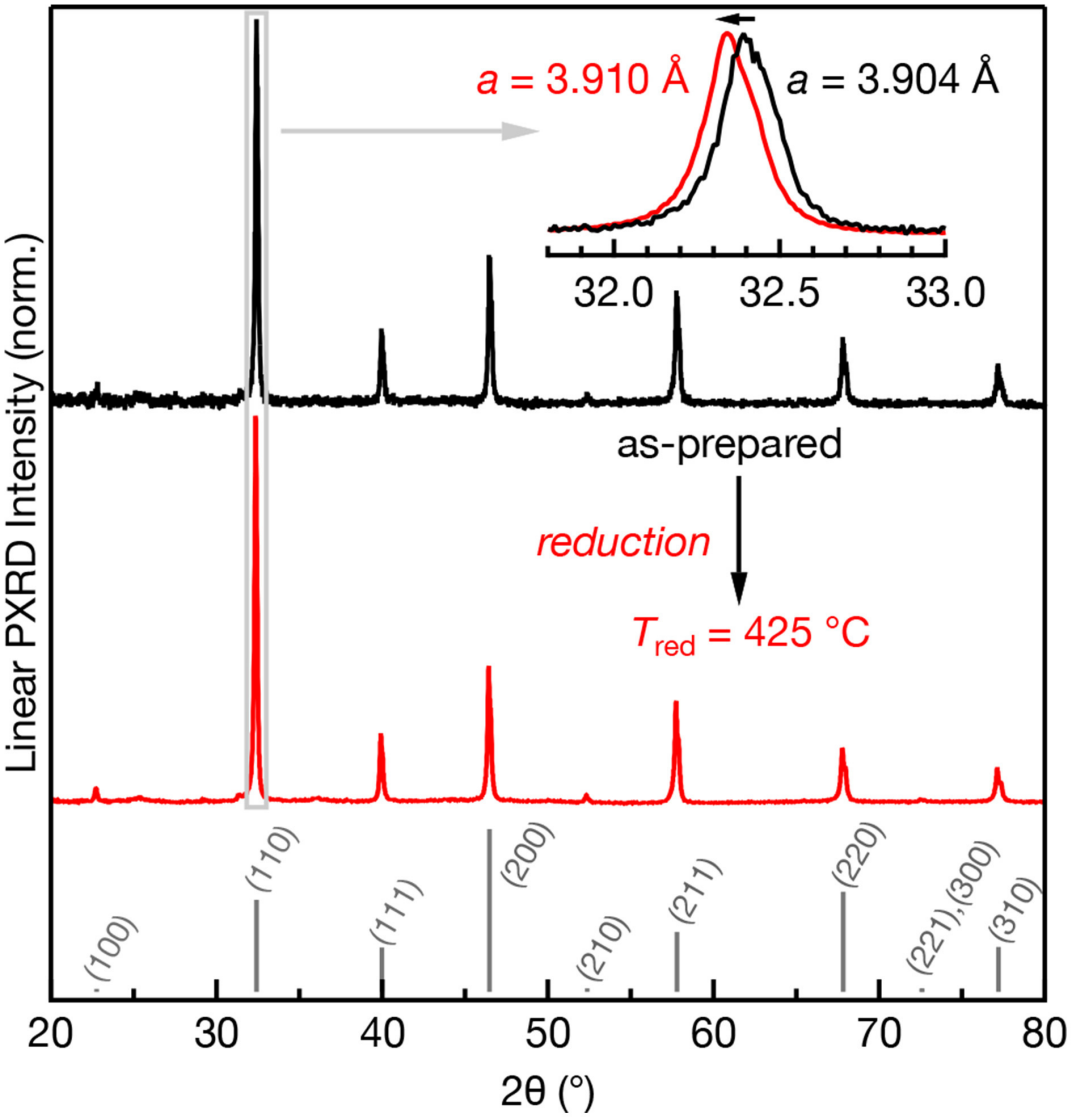
Powder XRD patterns of 0.1% Mn:SrTiO_3_ before (black) and after *T*_red_ = 425°C (red). The calculated pattern for SrTiO_3_ (cubic phase) is shown in gray as sticks (Yamanaka et al., 2002). Inset: expanded region of the (110) diffraction peak clearly showing a shift to lower 2θ after *T*_red_ = 425°C.

The room temperature powder XRD patterns of as-prepared and reduced (*T*_red_ = 425°C) Mn:SrTiO_3_ are shown in Figure 1. All samples designated Mn:SrTiO_3_ contain nominally 0.1% Mn content. Both the as-prepared and reduced samples indicate the presence of the cubic phase of SrTiO_3_ (Mitchell et al., 2000). However, a clear *increase* in the lattice parameter is observed after reduction. This result is consistent with other observations and has been attributed to both changes in ionic size and electronic effects after reduction of the lattice (Janotti et al., 2012). For example, the reduction of both Mn ions (Mn^4+^ → Mn^2+^) and lattice cations (Ti^4+^ → Ti^3+^) would result in larger ions leading to lattice expansion (Shannon, 1976). No appreciable secondary phases were observed after low-temperature chemical reduction despite clear spectroscopic changes in the samples (*vide infra*).

**Figure 2:**
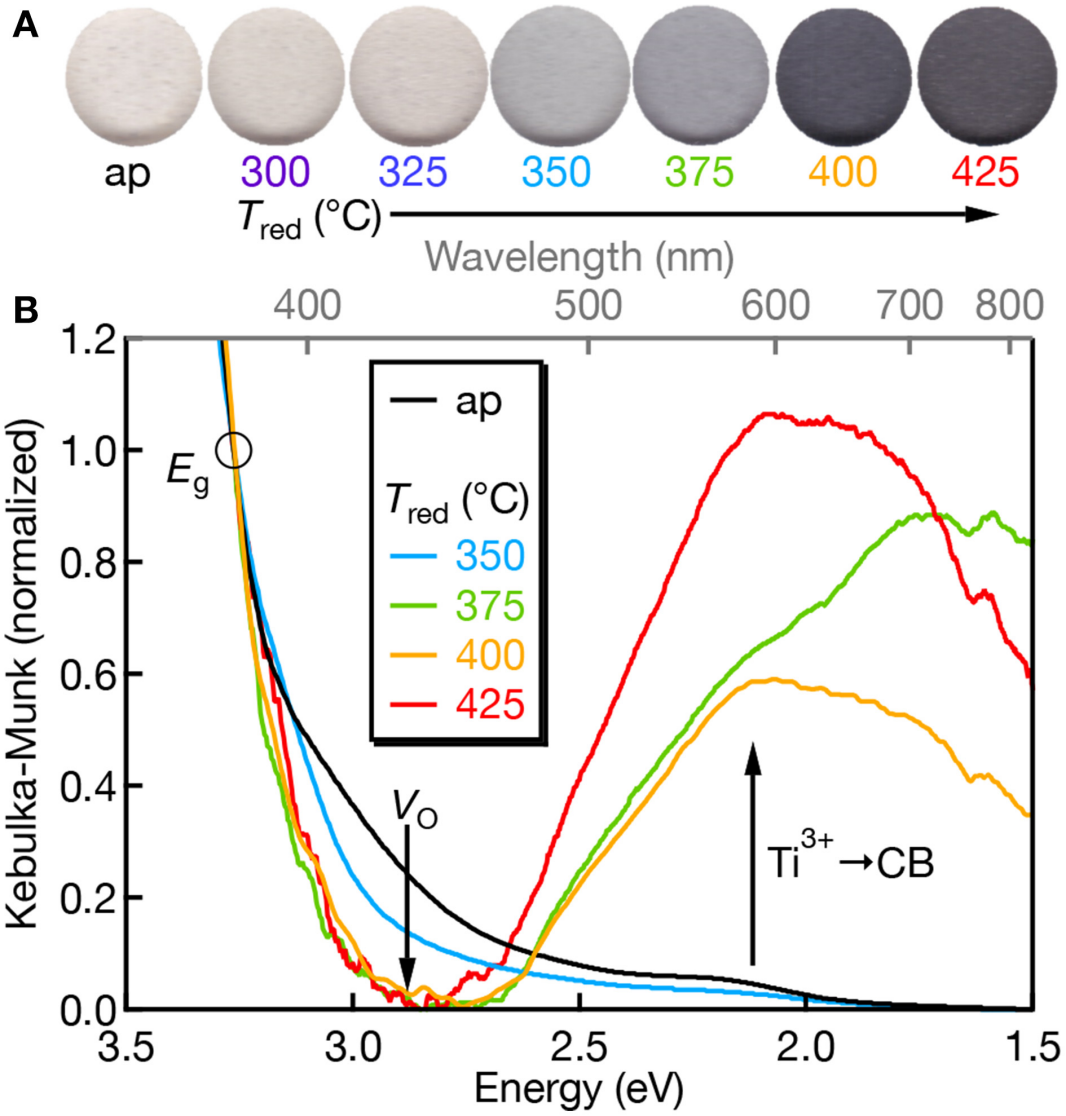
**(A)** Scanned color images of Mn:SrTiO_3_ powders as a function of *T*_red_. **(B)** Diffuse-reflectance spectra of Mn:SrTiO_3_ as a function of reduction temperature (as-prepared = black; *T*_red_ at 350°C (blue), 375°C (green), 400°C (orange), and 425°C (red), respectively). Data were normalized at 380 nm (3.26 eV; denoted by the circle) after setting the lowest y-value in the spectrum as the “zero.” The *T*_red_ = 400°C spectrum was smoothed for presentation.

The electronic structure of Mn:SrTiO_3_ is dependent on the nature of the Mn-ion speciation (i.e., oxidation state(s) and first-coordination sphere). Mn^4+^ has a d^3^ electronic configuration yielding a ^4^A_2g_ ground state when substituted at the Ti^4+^-site in SrTiO_3_. The physical appearance of the Mn:SrTiO_3_ as-prepared (oxidized) powders is off-white and gradually turns to black with increased reduction temperature as shown in Figure 2A. The black appearance of SrTiO_3_ has been previously observed and indicates reduction in the SrTiO_3_ lattice resulting in self-trapped electrons localized at Ti^3+^ sites (Tan et al., 2014; Lehuta and Kittilstved, 2016). The diffuse-reflectance spectra corroborates the assignment of the black color to excitations from Ti^3+^ to conduction band states also referred to as a metal-to-metal charge transfer (MMCT) transition in the near-IR region (Khomenko et al., 1998). In the Mn:SrTiO_3_ powder this MMCT appears at *T*_red_ between 350 and 375°C. The sub-bandgap tailing absorption at ~2.9 eV has been assigned to excitations from the valence band to *V*_O_'s with different charge states (Mitra et al., 2012). With increasing *T*_red_, the relative intensity of the *V*_O_-related transitions decreases and disappears at *T*_red_ = 375°C and is consistent with electron accumulation in the *V*_O_ states. Both spectral changes observed here for Mn:SrTiO_3_ with increasing *T*_red_ are similar to our recent study on chemically-reduced Cr:SrTiO_3_ (Lehuta and Kittilstved, 2016).

We also do not observe Mn-centered transitions from the diffuse-reflectance spectra which we attribute to either (1) low concentrations of Mn^3+^ or Mn^4+^, which have spin-allowed transitions in the visible, or (2) the Mn ions are primarily in their +2 oxidation state, which only has spin-forbidden transitions when the d-electrons order in the high-spin configuration (*S* = 5/2, ^6^A_1g_ ground state).

**Figure 3:**
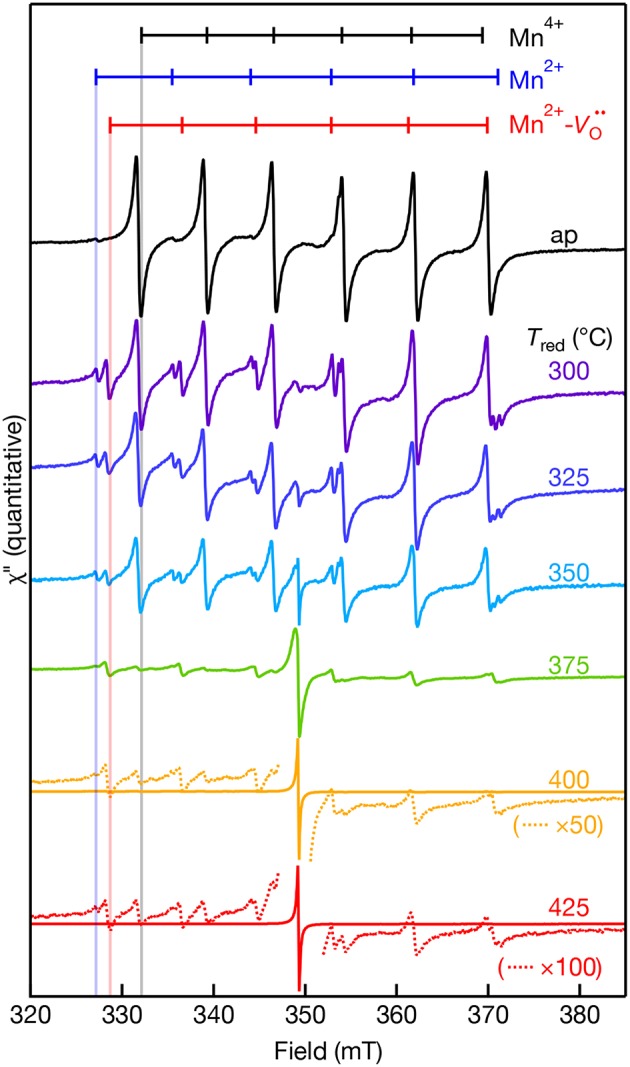
Room temperature X-band EPR spectra of 0.1% Mn-doped SrTiO_3_ bulk powder before (as-prepared, black) and after NaBH_4_ reduction for 30 min at various temperatures (300–425°C). The dotted lines in the 400 and 425°C spectra are selected regions of the spectra that are multiplied (×) by 50 and 100, respectively. The calculated resonance fields for the hyperfine-split transitions of Mn^4+^ (black), Mn^2+^-$V_{\text{o}}^{\cdot \cdot }$ (red), and Mn^2+^ (blue) in SrTiO_3_ are shown as vertical markers. The calculated resonance fields were simulated for each Mn center observed in the EPR with EasySpin (see characterization). Data were collected on the same day in a double-resonator cavity equipped with an as-prepared sample in the 2nd resonator and used to standardize sample intensities.

EPR-active species involving Mn ions in the +2, +3, and +4 oxidation states in SrTiO_3_ have previously been reported (Müller, 1959; Serway et al., 1977; Azamat et al., 2012). However, Mn^3+^ exhibits large zero-field splitting due to the *S* = 2 electronic spin state and thus, it is EPR-silent at conventional X-band frequencies (Azamat et al., 2012). The room temperature quantitative X-band EPR spectra of the Mn-doped SrTiO_3_ samples are shown in Figure 3 as a function of reduction temperatures ranging from *T*_red_ = 300–425°C. The as-prepared sample consists of two sets of sextet peaks. In accordance with the reported *g*-value and hyperfine splitting constant (*A*) of Mn^4+^ in SrTiO_3_, the main sextet in the as-prepared sample is assigned to Mn^4+^ substituting for Ti^4+^ with an isotropic *g* = 1.996 and |*A*| = 69.4 × 10^−4^ cm^−1^ (Müller, 1959). The second and much weaker set of sextets is somewhat occluded by the Mn^4+^ transitions, but the low-field resonances agree well with substitutional Mn^2+^ at the Ti^4+^ site in SrTiO_3_ with *g* = 2.004 and |*A*| = 82.30 × 10^−4^ cm^−1^ (Azzoni et al., 2000; Choudhury et al., 2013). Despite reports of both axial Mn^2+^-$V_{\text{o}}^{\cdot \cdot }$ and Mn^3+^-$V_{\text{o}}^{\cdot }$ complexes in Mn:SrTiO_3_, we do not observe these complexes in the as-prepared Mn:SrTiO_3_ sample. Hence, only the substitutional Mn^4+^ and Mn^2+^ species in an octahedral oxide crystal field co-exist in the as-prepared Mn:SrTiO_3_ powders.

After chemical reduction with NaBH_4_ under Ar(g) at *T*_red_ = 300°C, a new, third set of transitions are detected near the Mn^2+^ lines. Concomitant with the appearance of this new set of peaks is a decrease in the intensity of Mn^4+^ lines and an increase in the relative intensity of Mn^2+^. The new set of lines agree well with the report of a substitutional Mn^2+^ center at the Ti^4+^-site coupled to a doubly ionized oxygen vacancy (Serway et al., 1977). The reported EPR parameters of this Mn^2+^-$V_{\text{o}}^{\cdot \cdot }$ complex includes a large axial component to the zero-field splitting (*D* = 0.544 cm^−1^), *g*_||_ = 2.003, and |*A*| = 76 × 10^−4^ cm^−1^. We were unable to detect any transitions at lower or higher magnetic fields likely from the low relative concentration and low nominal concentration of Mn in the lattice. The Mn^2+^-$V_{\text{o}}^{\cdot \cdot }$ complex forms at low temperatures before reduction of the SrTiO_3_ lattice at *T*_red_ < 375°C (see Figure 2). One possible mechanism to explain the formation of this complex could be that oxygen vacancies may diffuse through the lattice and localize in the vicinity of Mn^4+^ substitutional sites at low temperatures. This work demonstrates that mild reduction at only *T*_red_ = 300–325°C is sufficient to form the Mn^2+^-$V_{\text{o}}^{\cdot \cdot }$ complex in bulk powders. This result contrasts with the high temperature reductions above 825°C previously used to create these centers in Mn:SrTiO_3_ (Blazey et al., 1983; Kutty et al., 1986). In addition, we observe the co-existence of Mn^4+^ and the Mn^2+^-$V_{\text{o}}^{\cdot \cdot }$ complex in the same EPR spectra at every *T*_red_. This does not agree with previous single crystal studies where the Mn^2+^-$V_{\text{o}}^{\cdot \cdot }$ complex was only observed when the Mn^4+^ lines were fully removed upon reduction in 5% hydrogen for 3 h at 1,000°C (Serway et al., 1977).

A new single feature centered at *B*_0_ ~ 350 mT (*g* ~ 2.003) with no associated hyperfine structure was also observed in the EPR spectra after reduction. This feature increases in spectral intensity and also narrows with increasing *T*_red_. This feature is similar to a feature observed in Cr:SrTiO_3_ at *T*_red_ > 375°C (Lehuta and Kittilstved, 2016), but has a significantly larger relative intensity compared to the dopant EPR signal in the Mn:SrTiO_3_ sample with the same nominal dopant concentration. This feature is tentatively assigned as the EPR-active superoxide anion (${\text{O}}_{2}^{-}$) adsorbed on the surface of SrTiO_3_ and could form via a surface reaction between Ti^3+^ and oxygen (Bykov et al., 2013; Harrigan et al., 2016). Table 1 below summarizes the EPR spectral parameters for previously reported Mn species in SrTiO_3_ in the as-prepared and reduced Mn:SrTiO_3_.

**Table 1:** Summary of EPR parameters for the Mn centers observed in the as-prepared and chemically treated samples reduced at various low temperatures.

**EPR center**	**Observed in this work**	***g*-value**	**|*A*| (x10^**−4**^ cm^**−1**^)**	**References**
Mn^4+^	As-prepared (strong) reduced (weak)	1.994	69.4	Müller, 1959
Mn^2+^	As-prepared (weak) reduced (strong)	2.004	82.3	Azzoni et al., 2000
*^a^*Mn^2+^-$V_{\text{o}}^{\cdot \cdot }$	Reduced (weak)	*g*_||_ = 2.003	76	Serway et al., 1977
Mn^3+^-$V_{\text{o}}^{\cdot }$	Not observed	*g*_||_ = 7.945 *g*_⊥_ < 0.4	37.3	Serway et al., 1977
${\text{O}}_{2}^{-}$	Reduced (strong)	2.003	–	this work

The g-values and |A| obtained in this work are corroborated with previous reports in the literature.

a *An additional large axial component to the zero-field splitting was also estimated, D = 0.544 cm^−1^*.

**Figure 4:**
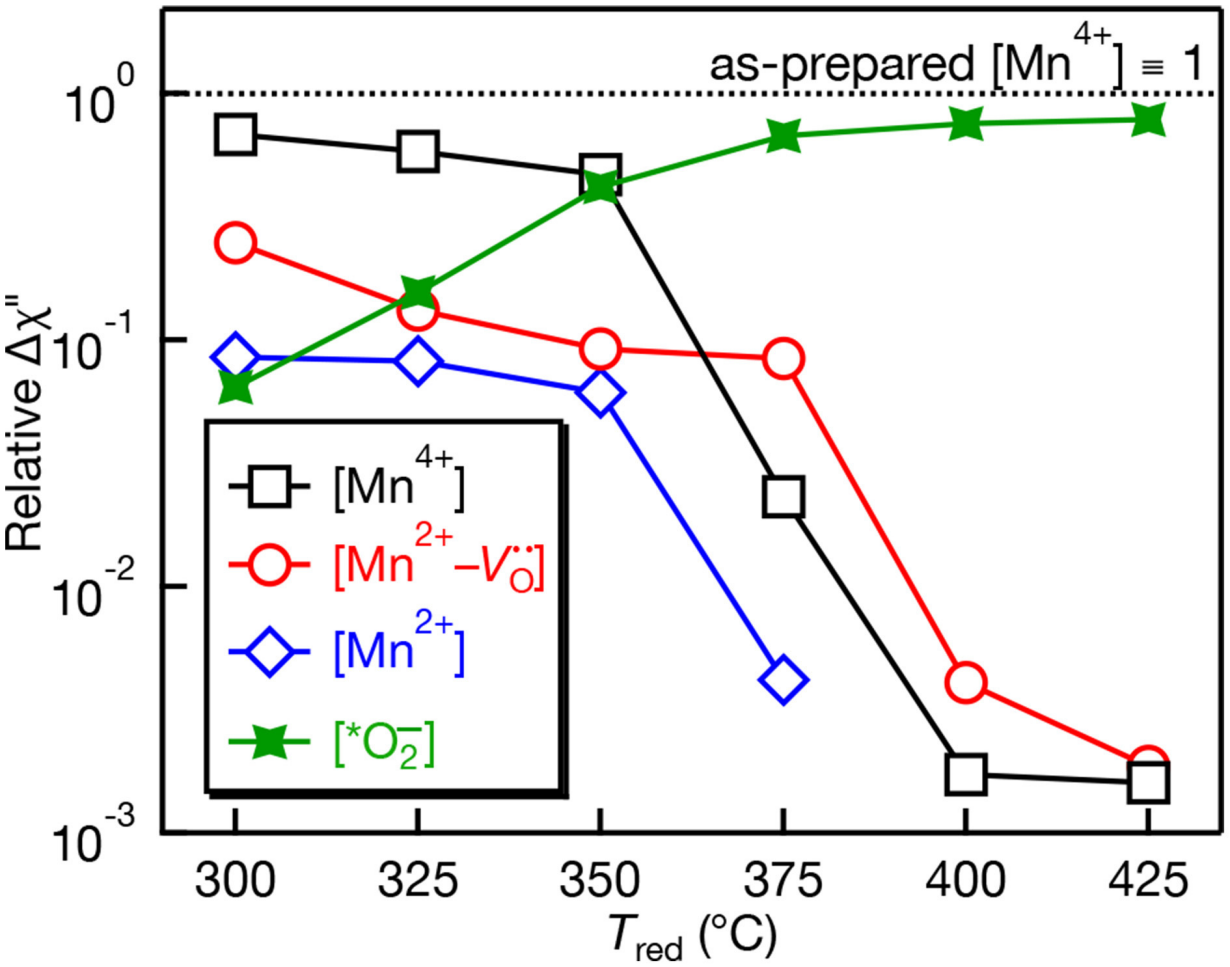
Relative EPR peak intensities plotted on a log scale as a function of NaBH_4_ reduction temperature (*T*_red_) for Mn:SrTiO_3_. The peak intensities are normalized vs. the intensity of the Mn^4+^ EPR signal in the as-prepared sample. Quantitative measurements performed using a dual-cavity EPR resonator (see characterization section).

The quantitative EPR spectra measured using the double resonator cavity shown in Figure 3 was analyzed and the relative intensity of the observed EPR centers is shown in Figure 4 as a function of *T*_red_. Compared to the as-prepared Mn^4+^ signal intensity (defined as 1), a gradual decrease in the Mn^4+^ and Mn^2+^ EPR signals is observed with similar correlations in their temperature dependences. The signal for Mn^2+^ is not detected for *T*_red_ ≥ 375°C. In contrast, the EPR intensity of the Mn^2+^-$V_{\text{o}}^{\cdot \cdot }$ complex shows little change and is more intense than the Mn^4+^ and Mn^2+^ EPR signals for *T*_red_ ≥ 375°C. The intensity of the Mn^2+^-$V_{\text{o}}^{\cdot \cdot }$ complex drops by an order of magnitude after increasing *T*_red_ from 375 to 400°C. At the highest temperature, *T*_red_ = 425°C, the EPR intensity of the ${\text{O}}_{2}^{-}$ ion is nearly 3 orders of magnitude more intense than the Mn^2+^-$V_{\text{o}}^{\cdot \cdot }$ complex, indicative of substantial surface defects. Studies to identify the nature of this defect center are currently underway.

**Figure 5:**
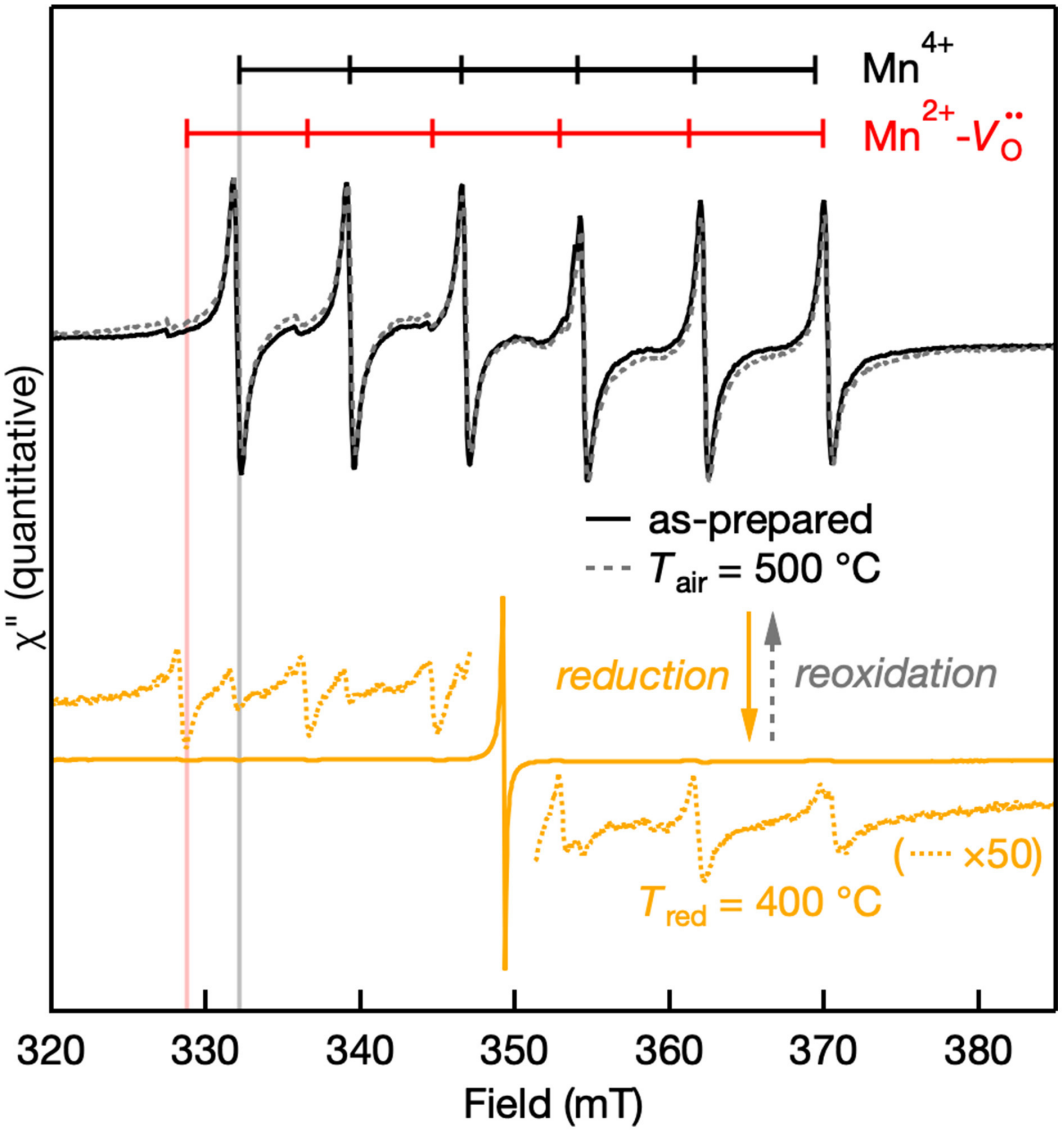
Quantitative reversibility of the EPR signal of Mn:SrTiO_3_ bulk powder as-prepared (solid black line), after *T*_red_ at 400°C (solid orange line), and after *T*_air_ at 500°C for ~1 h (gray dotted line). The weak Mn^4+^ and Mn^2+^ features after NaBH_4_ reduction are still observed in the 50× scaled spectra (orange dotted lines).

The EPR spectra of the Mn:SrTiO_3_ powders after preparation, after *T*_red_ = 400°C, and after aerobic reoxidation at *T*_air_ = 500°C for ~1 h are shown in Figure 5. The observed changes in the EPR spectra of the reduced samples revert to the as-prepared EPR spectrum by aerobically annealing the sample. The process of forming Mn^2+^-$V_{\text{o}}^{\cdot \cdot }$ complex in the reduced samples is thus reversible. However, elevated temperatures and longer reoxidation times were required in contrast with the chemical reductions. Since the Mn^2+^-$V_{\text{o}}^{\cdot \cdot }$ complex is a charge-neutral complex in the lattice, it is expected to be at least metastable. The apparently slower reoxidation kinetics compared to reduction kinetics suggest a metastable complex.

**Figure 6:**
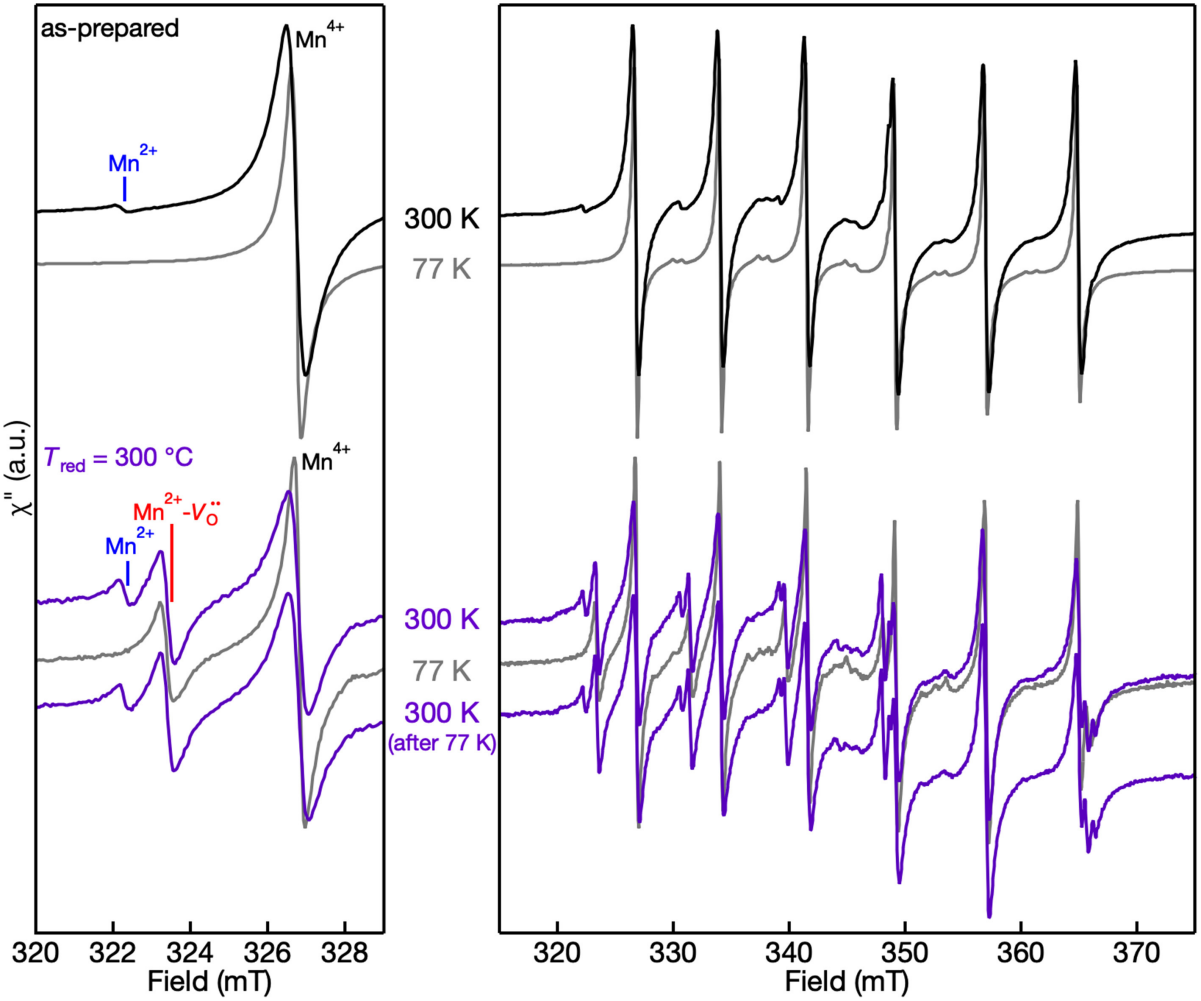
300 K and 77 K EPR spectra of Mn:SrTiO_3_ powders as-prepared **(top)** and after *T*_red_ = 300°C **(bottom)**. **(left)** Spectra in the region of the lowest field transitions within the hyperfine structure and **(right)** full spectrum. The temperatures at which the spectra were collected are labeled in the figure. Spectra are normalized to account for the increased EPR intensity with decreasing temperature.

The 300 K (room temperature) and 77 K (liquid N_2_) EPR spectra of the Mn centers in the as-prepared and *T*_red_ = 300°C powders are shown in Figure 6. Two things are revealed from the EPR spectra of both as-prepared and lightly-reduced Mn:SrTiO_3_ samples: (1) there is no evidence of self-trapped electrons at Ti^3+^ sites in the lattice based on the 77 K spectra, and (2) the EPR intensity of Mn^2+^ completely disappears at 77 K. At low temperature, the intensity of Mn^4+^ is pronounced following the typical Boltzmann statistics. In contrast, the Mn^2+^ EPR signal completely disappears at 77 K in these two samples. These results agree with a previous magnetic susceptibility and EPR study of the Mn^2+^ signal vanishing, where the behavior was attributed to increased antiferromagnetic interactions between adjacent Mn^2+^ ions with decreasing temperature (Azzoni et al., 2000). This explanation cannot be extended to describe the EPR signal of the Mn^2+^-$V_{\text{o}}^{\cdot \cdot }$ complex, which does not disappear at 77 K in the *T*_red_ = 300°C sample. To confirm the behavior of EPR signals as a function of temperature, the EPR spectrum of the reduced sample at 300 K was repeated after cooling it to 77 K and the entire spectrum is nearly identical.

**Figure 7:**
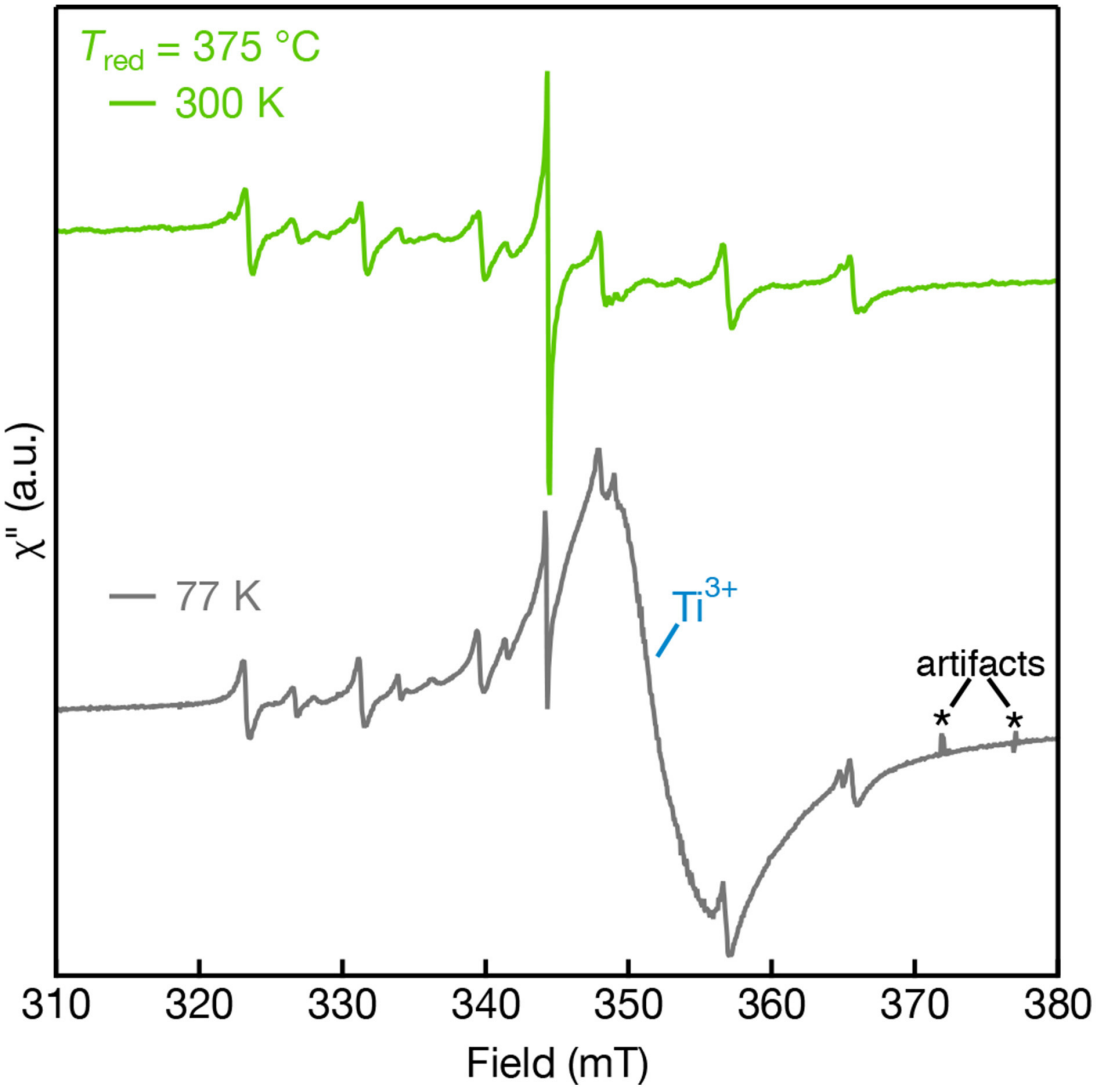
EPR spectra of Mn:SrTiO_3_ bulk powders reduced at *T*_red_ = 375°C measured at 300 K (top) and 77 K (bottom). Sharp features in the 77 K spectra are attributed to artifacts resulting from bubbles that arise in the liquid nitrogen finger dewar.

The cryogenic EPR measurements were also collected for samples reduced above 350°C to reveal the effect of Ti^3+^ defects on the EPR spectra that are observed in the diffuse-reflectance spectra shown in Figure 2. Figure 7 shows the 300 K and 77 K EPR spectra of *T*_red_ = 375°C. The paramagnetic Ti^3+^ defects are not observed in the EPR performed at 300 K due to fast spin-lattice relaxation but are promptly observed at 77 K (Lehuta and Kittilstved, 2016; Harrigan and Kittilstved, 2018). At 77 K, the *T*_red_ = 375°C sample is dominated by a broad and intense asymmetric Ti^3+^ lattice defect centered at *g* = 1.94. The appearance of this fast-relaxing defect, however, has no apparent effect on the linewidth of the Mn-centers nor the single line that we tentatively assign to surface-adsorbed ${\text{O}}_{2}^{-}$ ions. We recently showed that linewidth and relaxation-dynamics of substitutional Cr^3+^ ions in SrTiO_3_ powders and colloidal nanocrystals can be significantly altered when Ti^3+^ defects are present in the lattice through a near-resonant cross-relaxation process (Lehuta and Kittilstved, 2016; Harrigan and Kittilstved, 2018). This same behavior is not observed for any of the Mn-centers in the reduced SrTiO_3_ powder.

## Conclusions

A low-temperature chemical reduction technique has been implemented for tunability of the Mn dopant oxidation states and the related intrinsic defects in bulk Mn:SrTiO_3_. We employed a myriad of structural and spectroscopic techniques on samples subjected to a systematic chemical reduction. Both isotropic Mn^4+^ and Mn^2+^ species were identified in the as-prepared powders. Following the thermal reduction, the samples exhibited a continuous decrease in Mn^4+^ EPR signal and an increase in the Mn^2+^ intensity, accompanied by the introduction of a Mn^2+^-$V_{\text{o}}^{\cdot \cdot }$ complex. We demonstrate that our chemical treatment at merely *T*_red_ = 300–325°C generates sufficient driving force to significantly reduce the intensity of the octahedral Mn^4+^ and Mn^2+^ dopants and form the Mn^2+^-$V_{\text{o}}^{\cdot \cdot }$ complex. All the Mn peaks showed distinctive changes at low-temperature in the EPR that are readily reversible upon warming back the samples. Reductions at 375°C and above generated significant concentrations of Ti^3+^ defects that were confirmed by diffuse-reflectance and low-temperature EPR spectroscopy. All the observed perturbations in the reduced samples are entirely reversible by aerobic annealing at elevated temperatures. We also observe an intense spectral feature in the EPR spectrum in heavily-reduced Mn:SrTiO_3_ powders that we attribute to ${\text{O}}_{2}^{-}$ ions at the surface. This fast and effective strategy offers a general low-temperature reduction process that allows tunability and control over the rich dopant-defect chemistry in transition-metal doped SrTiO_3_ materials.

## Data Availability

The datasets generated for this study are available on request to the corresponding author.

## Author Contributions

HM and KL carried out the experiments, data analysis and interpretation, and edited the manuscript. WH contributed to the interpretation of the results and edited the manuscript. HM and KK contributed to the data analysis and interpretation and wrote the manuscript.

### Conflict of Interest Statement

The authors declare that the research was conducted in the absence of any commercial or financial relationships that could be construed as a potential conflict of interest.

## References

[B1] Azamat D. V. Badalyan A. G. Dejneka A. Trepakov V. A. Jastrabik L. Frait Z. (2012). High-frequency electron paramagnetic resonance investigation of Mn^3+^ centers in SrTiO_3_. J. Phys. Chem. Solids 73, 822–826. 10.1016/j.jpcs.2012.02.009

[B2] Azzoni C. B. Mozzati M. C. Paleari A. Massarotti V. Bini M. Capsoni D. (2000). Magnetic evidence of different environments of manganese ions in Mn-substituted strontium titanate. Solid State Commun. 114, 617–622. 10.1016/s0038-1098(00)00121-6

[B3] Blazey K. W. Cabrera J. M. Müller K. A. (1983). Oxygen vacancy-transition metal-ion impurity association in SrTiO_3_. Solid State Commun. 45, 903–906. 10.1016/0038-1098(83)90332-0

[B4] Bykov I. Makarova M. Trepakov V. Dejneka A. Yurchenko L. Yurchenko L. (2013). Intrinsic and impurity defects in chromium-doped SrTiO_3_ nanopowders: EPR and NMR study. Phys. Status Solidi B 250, 821–824. 10.1002/pssb.201200871

[B5] Chan N.-H. Sharma R. K. Smyth D. M. (1981). Nonstoichiometry in SrTiO_3_. J. Electrochem. Soc. 128, 1762–1769. 10.1149/1.2127727

[B6] Choudhury D. Mukherjee S. Mandal P. Sundaresan A. Waghmare U. V. Bhattacharjee S. (2011). Tuning of dielectric properties and magnetism of SrTiO_3_ by site-specific doping of Mn. Phys. Rev. B 84:125124 10.1103/PhysRevB.84.125124

[B7] Choudhury D. Pal B. Sharma A. Bhat S. V. Sarma D. D. (2013). Magnetization in electron- and Mn-doped SrTiO_3_. Sci. Rep. 3:1433. 10.1038/srep01433 23478593 PMC3594752

[B8] Eaton G. R. Eaton S. S. Barr D. P. Weber R. T. (2010). Quantitative EPR. Vienna: Spring-Verlag/Wien 10.1007/978-3-211-92948-3

[B9] Faughnan B. W. (1971). Photochromism in transition-metal-doped SrTiO_3_. Phys. Rev. B 4, 3623–3636. 10.1103/PhysRevB.4.3623

[B10] Harrigan W. L. Kittilstved K. R. (2018). Reversible modulation of the Cr^3+^ spin dynamics in colloidal SrTiO_3_ nanocrystals. J. Phys. Chem. C 122, 26652–26657. 10.1021/acs.jpcc.8b08680

[B11] Harrigan W. L. Michaud S. E. Lehuta K. A. Kittilstved K. R. (2016). Tunable electronic structure and surface defects in chromium-doped colloidal SrTiO_3−δ_ nanocrystals. Chem. Mater. 28, 430–433. 10.1021/acs.chemmater.6b00049

[B12] Ishii T. Kato H. Kudo A. (2004). H_2_ evolution from an aqueous methanol solution on SrTiO_3_ photocatalysts codoped with chromium and tantalum ions under visible light irradiation. J. Photochem. Photobiol. A Chem. 163, 181–186. 10.1016/S1010-6030(03)00442-8

[B13] Janotti A. Jalan B. Stemmer S. Van de Walle C. G. (2012). Effects of doping on the lattice parameter of SrTiO_3_. Appl. Phys. Lett. 100:262104 10.1063/1.4730998

[B14] Kamalasanan M. N. Deepak Kumar N. Chandra S. (1993). Structural, optical, and dielectric properties of sol-gel derived SrTiO_3_ thin films. J. Appl. Phys. 74, 679–686. 10.1063/1.355230

[B15] Kato H. Kudo A. (2002). Visible-light-response and photocatalytic activities of TiO_2_ and SrTiO_3_ photocatalysts codoped with antimony and chromium. J. Phys. Chem. B 106, 5029–5034. 10.1021/jp0255482

[B16] Kato H. Sasaki Y. Shirakura N. Kudo A. (2013). Synthesis of highly active rhodium-doped SrTiO_3_ powders in Z-scheme systems for visible-light-driven photocatalytic overall water splitting. J. Mater. Chem. A 1, 12327–12333. 10.1039/c3ta12803b

[B17] Khomenko V. M. Langer K. Rager H. Fett A. (1998). Electronic absorption by Ti^3+^ ions and electron delocalization in synthetic blue rutile. Phys. Chem. Miner. 25, 338–346. 10.1007/s002690050124

[B18] Kozuka Y. Hikita Y. Bell C. Hwang H. Y. (2010). Dramatic mobility enhancements in doped SrTiO_3_ thin films by defect management. Appl. Phys. Lett. 97:012107 10.1063/1.3457994

[B19] Kutty T. R. N. Gomathi Devi L. Murugaraj P. (1986). The change in oxidation state of Mn ions in semiconducting BaTiO_3_ and SrTiO_3_ around the phase transition temperatures. Mater. Res. Bull. 21, 1093–1102. 10.1016/0025-5408(86)90225-4

[B20] La Mattina F. Bednorz J. G. Alvarado S. F. Shengelaya A. Keller H. (2008). Detection of charge transfer processes in Cr-doped SrTiO_3_ single crystals. Appl. Phys. Lett. 93:022102 10.1063/1.2959059

[B21] Lehuta K. A. Haldar A. Zhou D. Kittilstved K. R. (2017). Spectroscopic study of the reversible chemical reduction and reoxidation of substitutional Cr ions in Sr_2_TiO_4_. Inorg. Chem. 56, 9177–9184. 10.1021/acs.inorgchem.7b01210 28714679

[B22] Lehuta K. A. Kittilstved K. R. (2016). Reversible control of the chromium valence in chemically reduced Cr-doped SrTiO_3_ bulk powders. Dalton. Trans. 45, 10034–10041. 10.1039/c6dt00706f 27117721

[B23] Mattheiss L. F. (1972). Effect of the 110°K Phase transition on the SrTiO_3_ conduction bands. Phys. Rev. B 6, 4740–4753. 10.1103/PhysRevB.6.4740

[B24] Middey S. Meneghini C. Ray S. (2012). Evidence of oxygen-vacancy-induced ferromagnetic order in single crystal Mn-doped SrTiO_3_. Appl. Phys. Lett. 101:042406 10.1063/1.4738785

[B25] Mitchell R. H. Chakhmouradian A. R. Woodward P. M. (2000). Crystal chemistry of perovskite-type compounds in the tausonite-loparite series, (Sr_1–2x_Na_x_La_x_)TiO_3_. Phys. Chem. Miner. 27, 583–589. 10.1007/s002690000103

[B26] Mitra C. Lin C. Robertson J. Demkov A. A. (2012). Electronic structure of oxygen vacancies in SrTiO_3_ and LaAlO_3_. Phys. Rev. B 86:155105 10.1103/PhysRevB.86.155105

[B27] Müller K. Burkard H. (1979). SrTiO_3_: an intrinsic quantum paraelectric below 4 K. Phys. Rev. B 19, 3593–3602. 10.1103/PhysRevB.19.3593

[B28] Müller K. A. (1959). Electron paramagnetic resonance of manganese IV in SrTiO_3_. Phys. Rev. Lett. 2, 341–343. 10.1103/PhysRevLett.2.341

[B29] Sasaki Y. Nemoto H. Saito K. Kudo A. (2009). Solar water splitting using powdered photocatalysts driven by Z-schematic interparticle electron transfer without an electron mediator. J. Phys. Chem. C 113, 17536–17542. 10.1021/jp907128k

[B30] Savinov M. Trepakov V. A. Syrnikov P. P. Železný V. Pokorný J. Dejneka A. (2008). Dielectric properties of Mn doped SrTiO_3_. J. Phys. Cond. Matter 20:095221 10.1088/0953-8984/20/9/095221

[B31] Serway R. A. Berlinger W. Müller K. A. Collins R. W. (1977). Electron paramagnetic resonance of three manganese centers in reduced SrTiO_3_. Phys. Rev. B 16, 4761–4768. 10.1103/PhysRevB.16.4761

[B32] Shannon R. D. (1976). Revised effective ionic radii and systematic studies of interatomic distances in halides and chalcogenides. Acta Cryst. A32, 751–767. 10.1107/S0567739476001551

[B33] Stoll S. Schweiger A. (2006). EasySpin, a comprehensive software package for spectral simulation and analysis in EPR. J. Magn. Reson. 178, 42–55. 10.1016/j.jmr.2005.08.013 16188474

[B34] Tan H. Zhao Z. Zhu W. B. Coker E. N. Li B. Zheng M. (2014). Oxygen vacancy enhanced photocatalytic activity of pervoskite SrTiO_3_. ACS Appl. Mater. Interfaces 6, 19184–19190. 10.1021/am5051907 25311356

[B35] Thanh T. D. Phan T. L. Oanh L. M. Minh N. V. Lee J. S. Yu S. C. (2014). Influence of Mn doping on the crystal structure, and optical and magnetic properties of SrTiO_3_ compounds. IEEE Trans. Magn. 50, 1–4. 10.1109/TMAG.2014.2304562

[B36] Wang D. Ye J. Kako T. Kimura T. (2006). Photophysical and photocatalytic properties of SrTiO_3_ doped with Cr cations on different sites. J. Phys. Chem. B 110, 15824–15830. 10.1021/jp062487p 16898732

[B37] Wang Q. Hisatomi T. Ma S. S. K. Li Y. Domen K. (2014). Core/shell structured La- and Rh-codoped SrTiO_3_ as a hydrogen evolution photocatalyst in Z-scheme overall water splitting under visible light irradiation. Chem. Mater. 26, 4144–4150. 10.1021/cm5011983

[B38] Weaver H. E. (1959). Dielectric properties of single crystals of SrTiO_3_ at low temperatures. J. Phys. Chem. Solids 11, 274–277. 10.1016/0022-3697(59)90226-4

[B39] Wild R. L. Rockar E. M. Smith J. C. (1973). Thermochromism and electrical conductivity in doped SrTiO_3_. Phys. Rev. B 8, 3828–3835. 10.1103/PhysRevB.8.3828

[B40] Yamanaka T. Hirai N. Komatsu Y. (2002). Structure change of Ca_1−x_Sr_x_TiO_3_ perovskite with composition and pressure. Am. Miner. 87, 1183–1189. 10.2138/am-2002-8-917

[B41] Zuo F. Wang L. Wu T. Zhang Z. Borchardt D. Feng P. (2010). Self-doped Ti^3+^ enhanced photocatalyst for hydrogen production under visible light. J. Am. Chem. Soc. 132, 11856–11857. 10.1021/ja103843d 20687606

